# Supplementary material for: Herbal Polyphenols as Selenium Reducers in the Green Synthesis of Selenium Nanoparticles: Antibacterial and Antioxidant Capabilities of the Obtained SeNPs
Source: Molecules. 2024 Apr 9;29(8):1686. doi: 10.3390/molecules29081686 (PMC11052002; doi:10.3390/molecules29081686)
Supplement: Supplementary file 1 [file molecules-29-01686-s001.zip › molecules-2945793-supplementary.pdf]

Supplementary material

Herbal polyphenols as selenium reducers in the green synthesis of selenium nanoparticles. Antibacterial and antioxidant capabilities of the obtained SeNPs

Aleksandra Sentkowska, Julia Konarska , Jakub Szmytke, Anna Grudniak

Table 1S Size and polydispersity index (PDI) of obtained SeNPs.

	Average size [nm]	PDI
**Blackberry**		
SeNPs11	96.8	0.150
SeNPs11H	157	0.187
SeNPs12	169	0.211
SeNPs12H	171	0.268
SeNPs13	175	0.309
SeNPs13H	182	0.341
**Hop**		
SeNPs11	84.9	0.109
SeNPs11H	120	0.115
SeNPs12	143	0.154
SeNPs12H	165	0.195
SeNPs13	170	0.322
SeNPs13H	175	0.271
**Lemon balm**		
SeNPs11	79.0	0.103
SeNPs11H	115	0.121
SeNPs12	85	0.111
SeNPs12H	158	0.210
SeNPs13	113	0.172
SeNPs13H	162	0.261
**Raspberry**		
SeNPs11	74.0	0.132
SeNPs11H	84.1	0.175
SeNPs12	109	0.184
SeNPs12H	167	0.190
SeNPs13	124	0.141
SeNPs13H	181	0.263
**Sage**		
SeNPs11	74.0	0.125
SeNPs11H	93.1	0.212
SeNPs12	164	0.190
SeNPs12H	173	0.231
SeNPs13	179	0.333
SeNPs13H	182	0.397

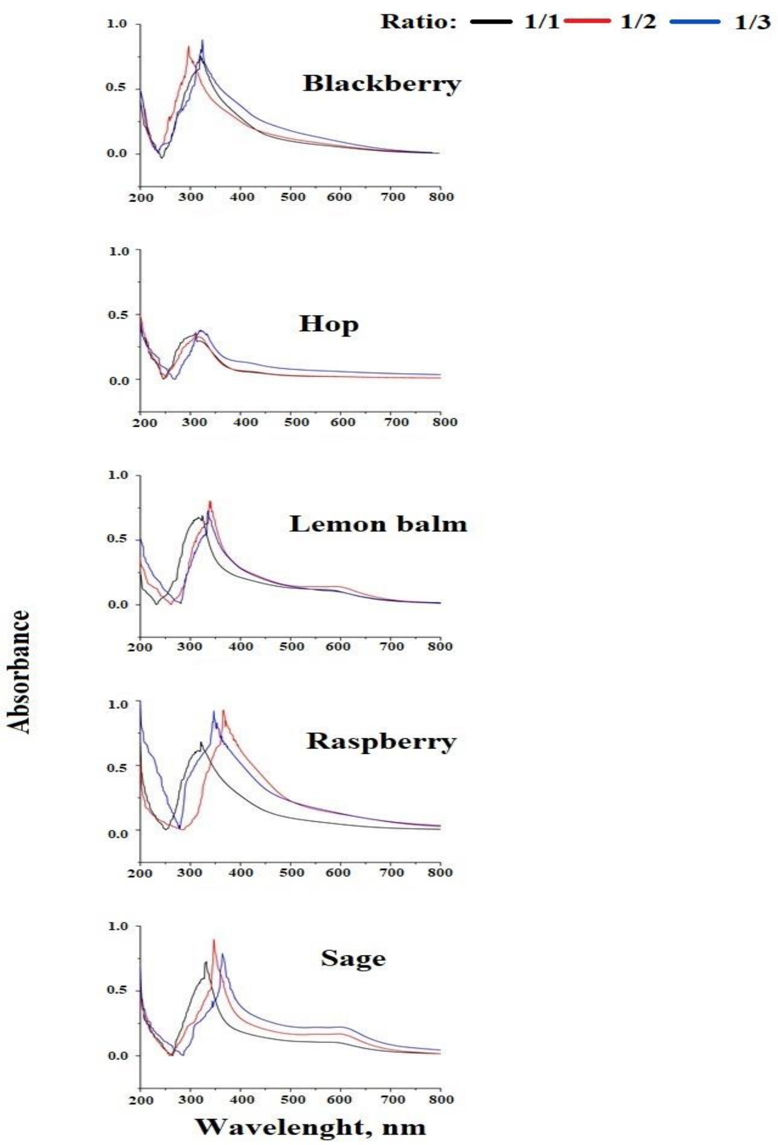

Fig 1S. UV-Vis spectra of synthesized SeNPs.

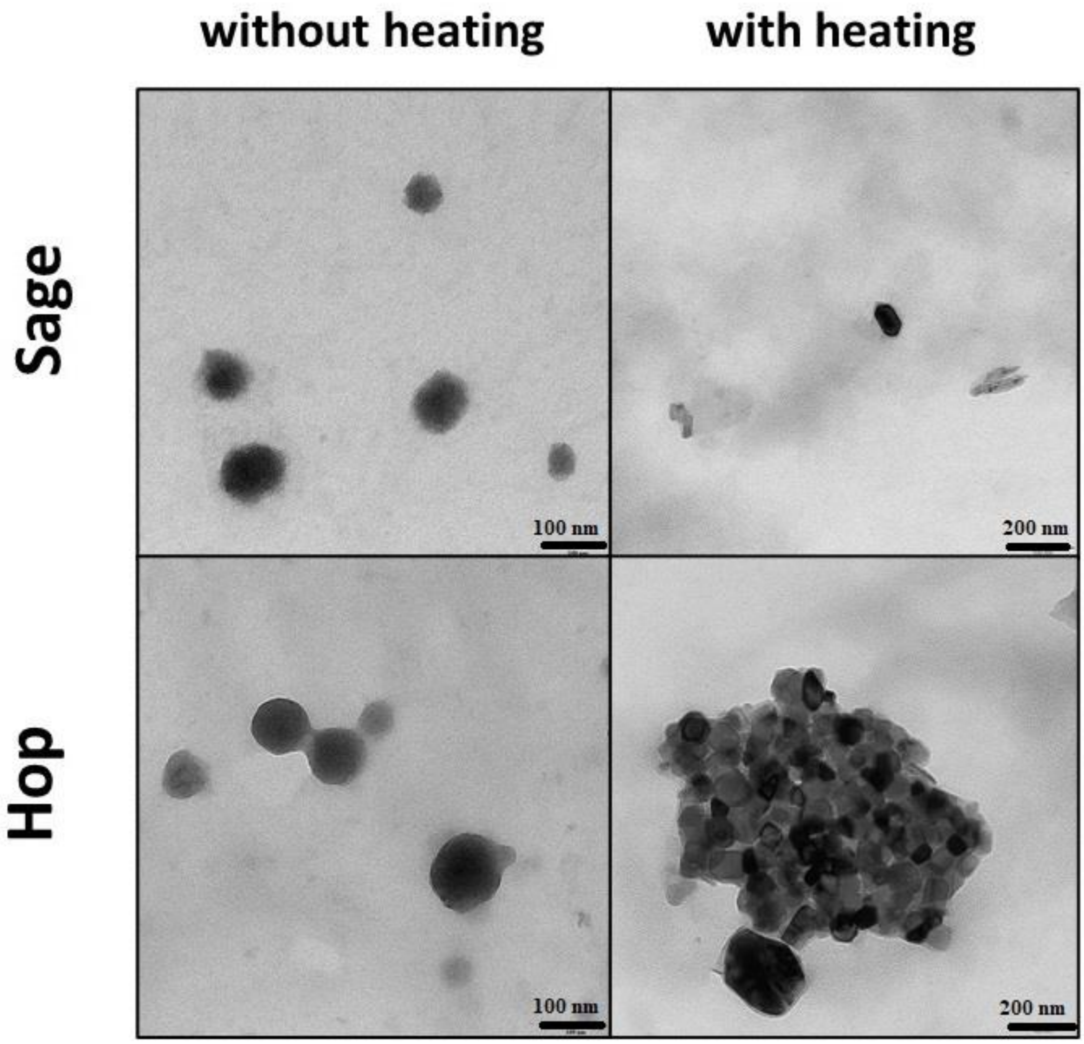

Fig 2S. The influence of post-reaction heating on shape and size of obtained SeNPs on the example of hop and sage.

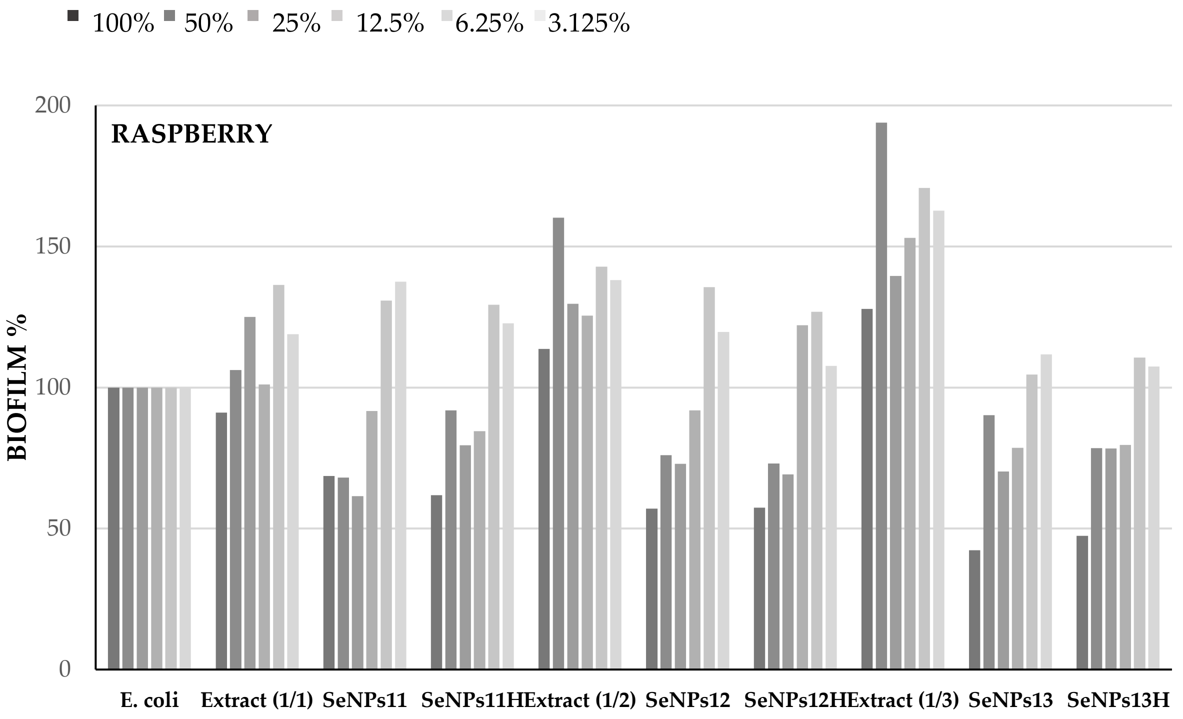
100%
50%
25%
12.5%
6.25%
3.125%
RASPBERRY
BIOFILM %
200
150
100
50
0
E. coli
Extract (1/1)
SeNPs11
SeNPs11H
Extract (1/2)
SeNPs12
SeNPs12H
Extract (1/3)
SeNPs13
SeNPs13H

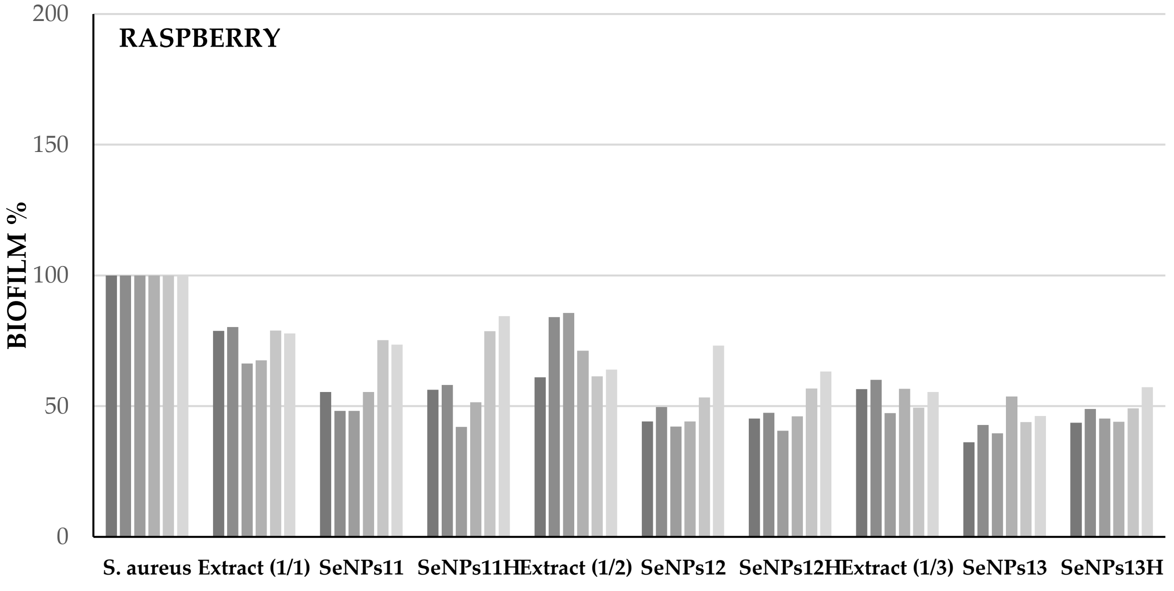
RASPBERRY
BIOFILM %
200
150
100
50
0
S. aureus
Extract (1/1)
SeNPs11
SeNPs11H
Extract (1/2)
SeNPs12
SeNPs12H
Extract (1/3)
SeNPs13
SeNPs13H

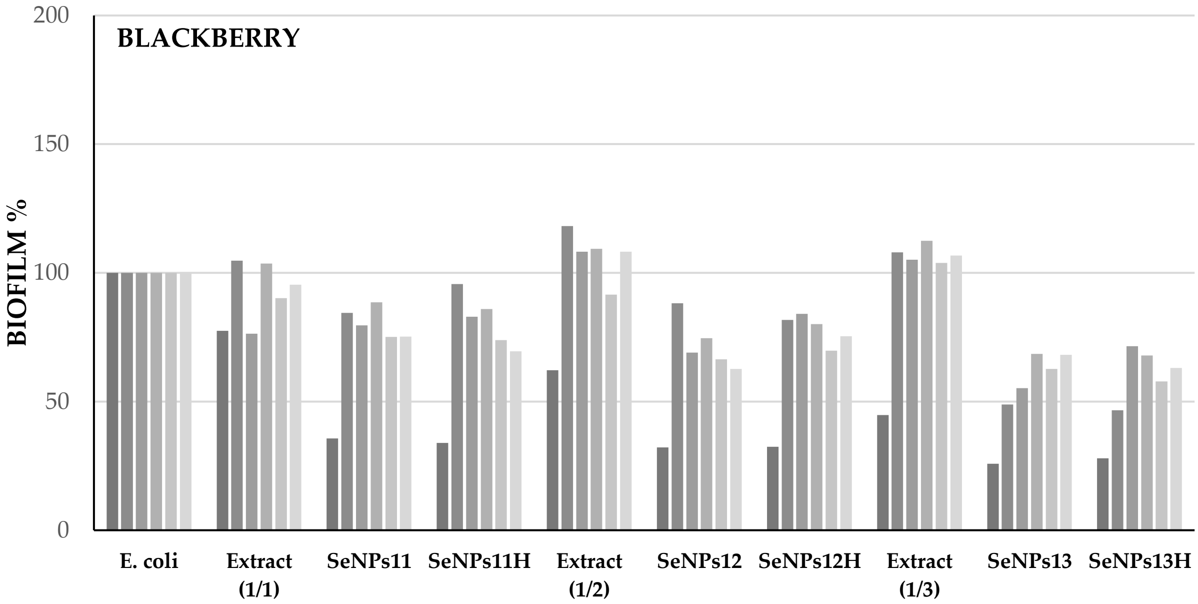
BLACKBERRY
BIOFILM %
200
150
100
50
0
E. coli
Extract (1/1)
SeNPs11
SeNPs11H
Extract (1/2)
SeNPs12
SeNPs12H
Extract (1/3)
SeNPs13
SeNPs13H

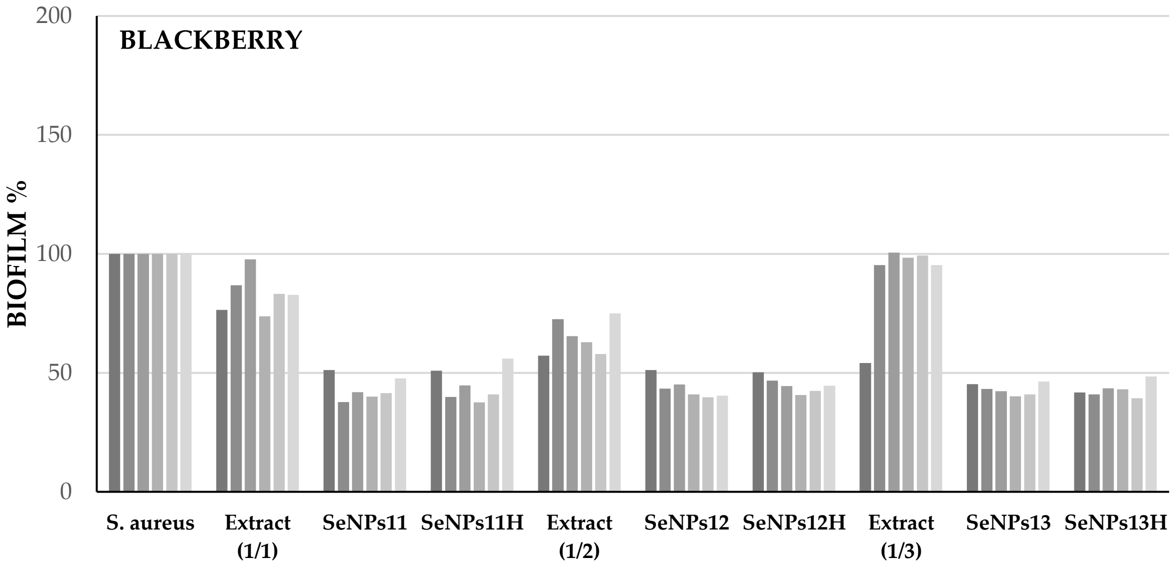
BLACKBERRY
BIOFILM %
200
150
100
50
0
S. aureus
Extract (1/1)
SeNPs11
SeNPs11H
Extract (1/2)
SeNPs12
SeNPs12H
Extract (1/3)
SeNPs13
SeNPs13H

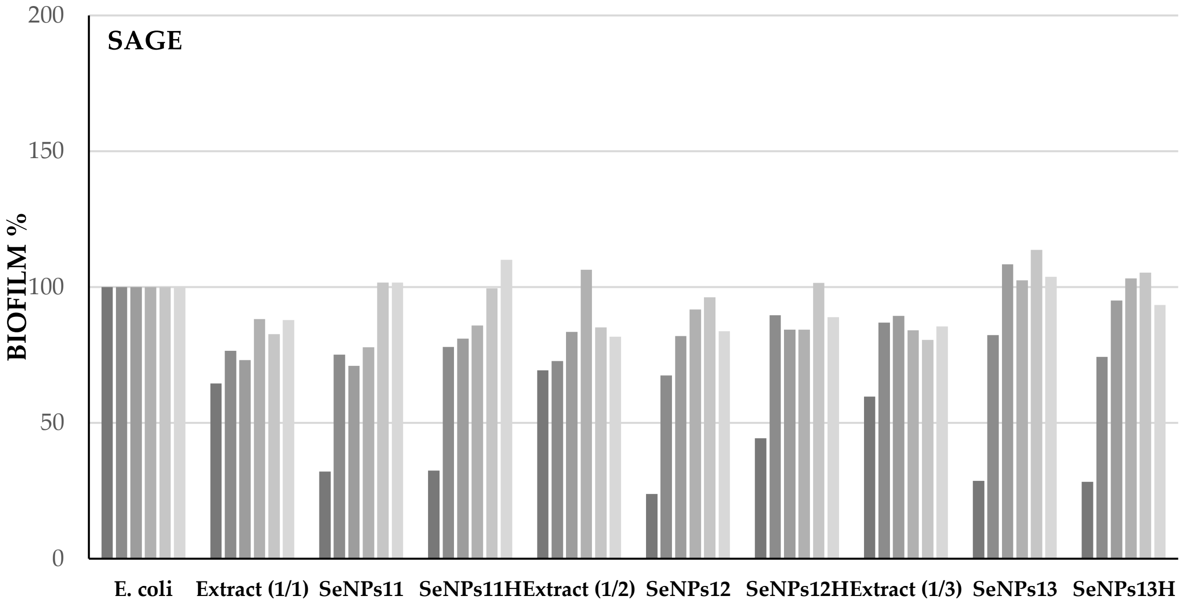
SAGE
BIOFILM %
200
150
100
50
0
E. coli
Extract (1/1)
SeNPs11
SeNPs11H
Extract (1/2)
SeNPs12
SeNPs12H
Extract (1/3)
SeNPs13
SeNPs13H

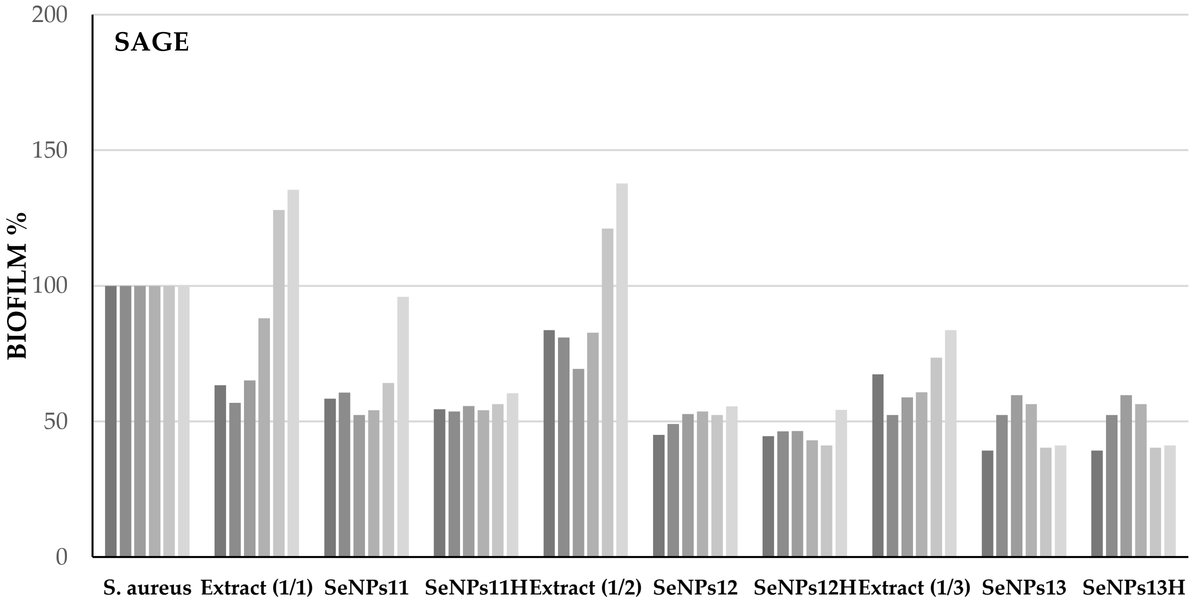
SAGE
BIOFILM %
200
150
100
50
0
S. aureus
Extract (1/1)
SeNPs11
SeNPs11H
Extract (1/2)
SeNPs12
SeNPs12H
Extract (1/3)
SeNPs13
SeNPs13H

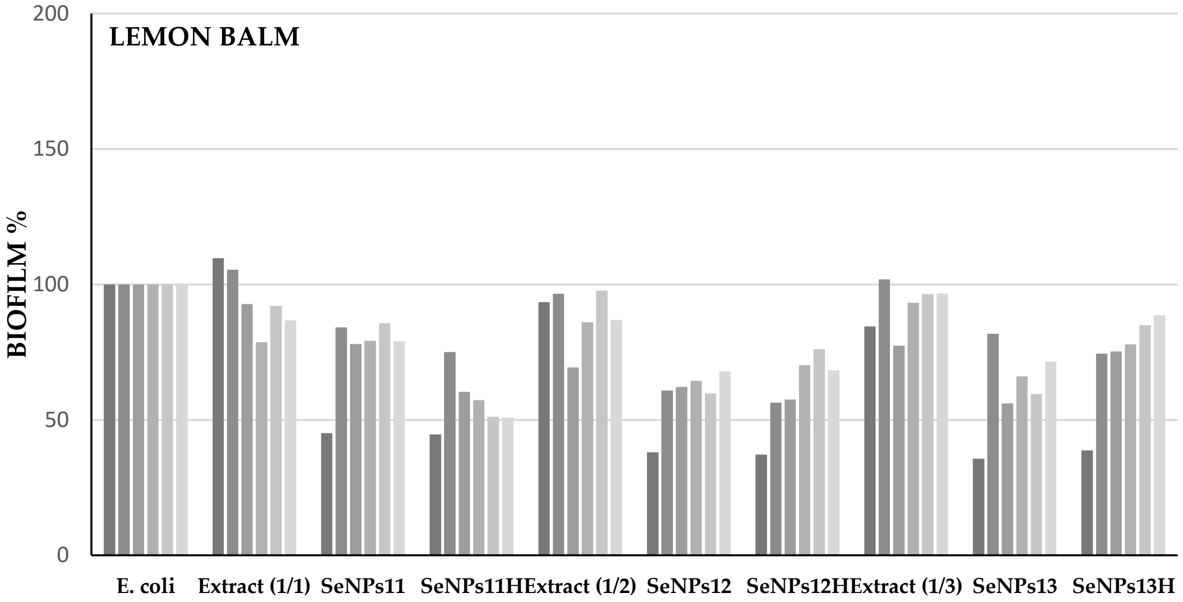
LEMON BALM
BIOFILM %
200
150
100
50
0
E. coli
Extract (1/1)
SeNPs11
SeNPs11H
Extract (1/2)
SeNPs12
SeNPs12H
Extract (1/3)
SeNPs13
SeNPs13H

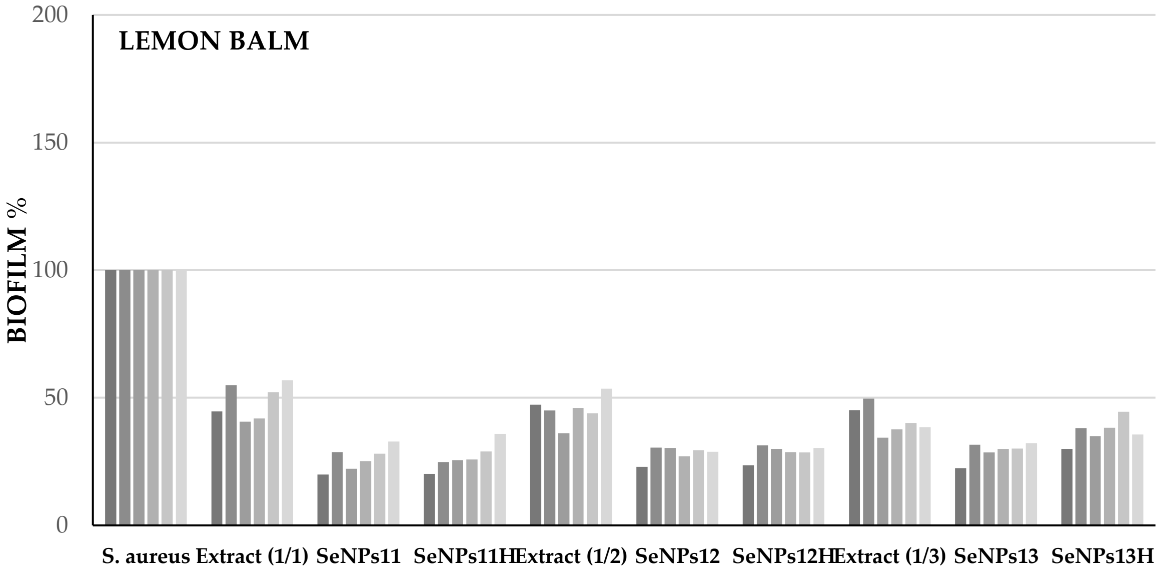
LEMON BALM
BIOFILM %
200
150
100
50
0
S. aureus
Extract (1/1)
SeNPs11
SeNPs11H
Extract (1/2)
SeNPs12
SeNPs12H
Extract (1/3)
SeNPs13
SeNPs13H

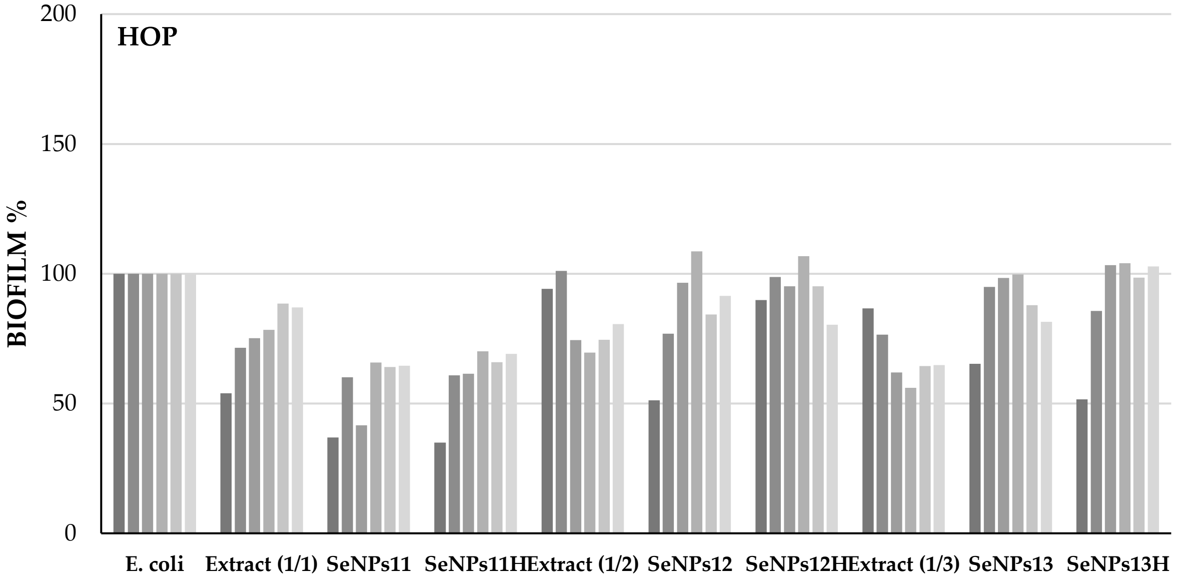
HOP
BIOFILM %
200
150
100
50
0
E. coli
Extract (1/1)
SeNPs11
SeNPs11H
Extract (1/2)
SeNPs12
SeNPs12H
Extract (1/3)
SeNPs13
SeNPs13H

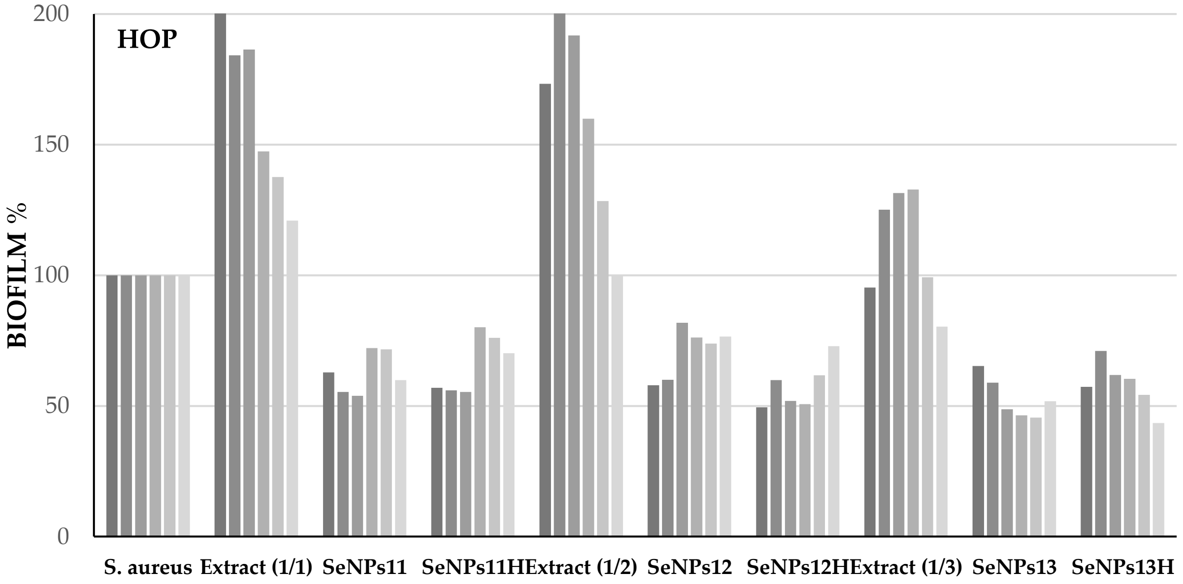
HOP
BIOFILM %
200
150
100
50
0
S. aureus
Extract (1/1)
SeNPs11
SeNPs11H
Extract (1/2)
SeNPs12
SeNPs12H
Extract (1/3)
SeNPs13
SeNPs13H

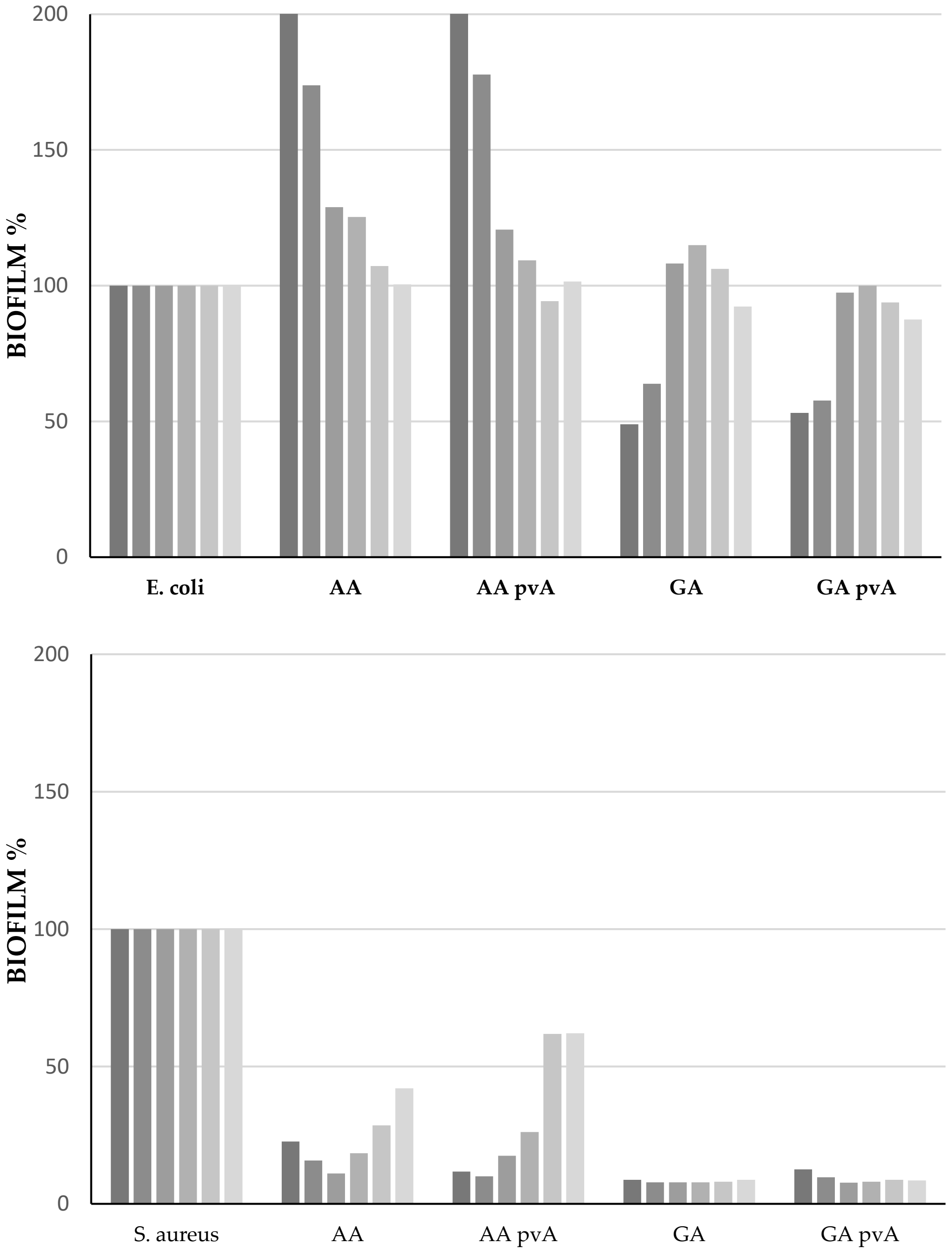

Fig 3S. The results of the biofilm analysis obtained with suspension of SeNPs against biofilms formed by *E. coli* and *S.aureus*.
